# Artificial Neural Network Inference (ANNI): A Study on Gene-Gene Interaction for Biomarkers in Childhood Sarcomas

**DOI:** 10.1371/journal.pone.0102483

**Published:** 2014-07-15

**Authors:** Dong Ling Tong, David J. Boocock, Gopal Krishna R. Dhondalay, Christophe Lemetre, Graham R. Ball

**Affiliations:** 1 The John van Geest Cancer Research Centre, School of Science and Technology, Nottingham Trent University, Nottingham, United Kingdom; 2 Imperial Centre for Translational and Experimental Medicine, National Heart and Lung Institute (NHLI), Imperial College London, London, United Kingdom; 3 Center for Molecular Oncology, Memorial Sloan Kettering Cancer Center, New York, New York, United States of America; University of Catania, Italy

## Abstract

**Objective:**

To model the potential interaction between previously identified biomarkers in children sarcomas using artificial neural network inference (ANNI).

**Method:**

To concisely demonstrate the biological interactions between correlated genes in an interaction network map, only 2 types of sarcomas in the children small round blue cell tumors (SRBCTs) dataset are discussed in this paper. A backpropagation neural network was used to model the potential interaction between genes. The prediction weights and signal directions were used to model the strengths of the interaction signals and the direction of the interaction link between genes. The ANN model was validated using Monte Carlo cross-validation to minimize the risk of over-fitting and to optimize generalization ability of the model.

**Results:**

Strong connection links on certain genes (TNNT1 and FNDC5 in rhabdomyosarcoma (RMS); FCGRT and OLFM1 in Ewing’s sarcoma (EWS)) suggested their potency as central hubs in the interconnection of genes with different functionalities. The results showed that the RMS patients in this dataset are likely to be congenital and at low risk of cardiomyopathy development. The EWS patients are likely to be complicated by EWS-FLI fusion and deficiency in various signaling pathways, including Wnt, Fas/Rho and intracellular oxygen.

**Conclusions:**

The ANN network inference approach and the examination of identified genes in the published literature within the context of the disease highlights the substantial influence of certain genes in sarcomas.

## Introduction

Although computer technologies have evolved over the past decades, debate regarding on the suitability of data mining techniques to identify “true” biomarkers continues due to the fact that these techniques are fundamentally based on mathematical paradigms. Furthermore, the connection between the statistical and the biological significance of the findings are not well described and validated. Questions regarding the advantage of these techniques and the relevance of the selected biomarkers to biological processes might explain why very few biomarkers that have been discovered using these approaches have seen clinical applications.

Additionally, many of the published studies assumed that biomarker discovery involves merely marker selection and classification. Markers with high statistical power and able to accurately predict the disease group are treated as “best” markers for clinical use, even though their biological interactivities are not tested *in silico*. This might explained why clinical trials on these markers have failed. We believe that the identification and validation of biomarkers *in silico* are equally important in biomarker discovery and are vital for clinical trial development. An *in silico* simulation of possible biological interaction between the selected candidate markers provides information on the nature of the markers (i.e. proactive or inactive), state of the markers (i.e. on or off) and possible chemical changes on the markers. These information can subsequently improve the success rate in clinical trials and patient care. In short, the biology of phenotype is more than just a list of markers; it is the complex interaction of biological components that defines phenotype.

We previously identified a list of high potential marker candidates that are able to differentiate small round blue cell tumors (SRBCTs) in children [1]. This study builds on previous work which has modeled the interaction between these markers to reveal their potential biological relevance in child sarcoma cancers using a bespoke artificial neural network based interactive algorithm.

The sarcoma groups in the SRBCTs dataset reported by Khan *et al*. [2] were used in this study. The selection of biomarker panels for the SRBCTs dataset was performed using a hybrid genetic algorithm-neural network (GANN) model, as has been reported in our previous work [1]. The aim of this GANN approach was to identify sets of features that possess significant statistics information and statistical comparison between classification methods based upon gene sets reported by Khan *et al*. and the GANN model has been elaborated. This study focused on modeling interactions between these features in order to infer their potential biological significance in sarcoma cancers.

It is difficult to objectively compare the biological relevance of individual genes identified between studies, as complex biology cannot be quantified using numerical analyses. Published literature [3], [4] has argued that the biological relevance between features can be achieved using correlation analysis and a pre-defined baseline value of the parameter which is known to be biologically meaningful. However, this information alone is not sufficient for comparing which feature has more biological meaning than another, as the full biological function of the features and the biochemical reaction mechanisms underlying regulatory interactions between features cannot be fully known without conducting a thorough assessment in clinical materials relevant to the disease status in order to describe behaviors of these features *in vivo*. This clinical assessment and validation is not within the remit of this paper and instead of looking for more biological meaningful features, this paper reports a complementary set of genes to those reported by Khan *et al* [2].

For the sake of conciseness of the interpretation of the biological relevance on the selected genes in the dataset, the biological functionality of the genes associating with 2 types of sarcomas, and interactions that are of potential relevance on the basis of plausible biological explanations and the correlation analysis of the genes were studied in this paper.

## Materials and Methods

In this section, we describe the SRBCTs dataset and the selected biomarkers by the GANN model. We then describe the framework of the interactome network analysis constructed using ANNs.

### Small round blue cell tumors (SRBCTs) dataset

The SRBCTs cDNA microarray dataset was originally studied by Khan *et al*. [2], with the scope of identifying marker genes that are able to distinguish 4 different types of blue cell tumors which often masquerade as one another in childhood. This was performed with ANN classification models in conjunction with Principle Component Analysis (PCA). The original dataset contained 88 samples with 2,308 genes, distributed into 4 different tumor types, *i.e.* rhabdomyosarcoma (RMS), Ewing’s sarcoma (EWS), Burkitt lymphoma (BL) and neuroblastoma (NB). Amongst the 88 samples, 25 were in the RMS group, 29 in EWS, 11 in BL, 18 in NB and the remaining 5 samples were unknown.

### Feature selection using genetic algorithm-neural network (GANN)

The GANN is a hybrid model which we have previously applied to the screening of datasets for genes of statistical relevance [1], [5]–[7]. In the GANN model, genetic algorithm (GA) and ANN co-evolve in the learning process. In brief, a population of chromosomes each representing a subset of microarray genes was first generated and the fitness for each chromosome was computed as a solution to the problem using multilayered ANNs. These fitness values were then iteratively evaluated using GA’s operators and ANNs, and a rank order of the genes based on their fitness values was produced. The evaluation process was iterated 3,000 times and the whole process cycle was repeated 5,000 times. The complete parameter settings on the GANN model can be found in our work [1].

The previously reported panel of 96 genes [1] is summarized in Table 1. Among these genes, 44 were complementary to the top 96 genes reported by Khan *et al*. [2].

**Table 1:** Summary of the previously selected 96 genes by GANN.

GANN rank	Symbol	Image Id.	GeneID	Truncated p-value for each cancer			
				EWS	BL	NB	RMS
1	MLLT11	812105	10962	8.65×10^−12^	4.12×10^−16^	7.27×10^−17^	1.33×10^−10^
2	IGF2	207274	3481	1.03×10^−07^	5.65×10^−11^	7.74×10^−06^	4.78×10^−09^
3	FCGRT	770394	2217	1.03×10^−13^	6.87×10^−13^	5.69×10^−10^	2.27×10^−07^
4	CAV1	377461	857	5.21×10^−11^	1.00×10^−10^	1.02×10^−08^	8.49×10^−08^
5	FGFR4	784224	2264	5.20×10^−08^	5.44×10^−08^	2.90×10^−08^	8.32×10^−11^
6	CD99	1435862	4267	8.12×10^−12^	6.96×10^−13^	8.43×10^−09^	1.04×10^−05^
7	IGF2	296448	3481	1.33×10^−07^	9.71×10^−10^	5.37×10^−05^	7.89×10^−09^
8	MAP1B	629896	4131	1.30×10^−05^	4.24×10^−11^	1.90×10^−07^	1.41×10^−04^
9	KDSR	814260	2531	2.11×10^−09^	2.49×10^−08^	1.13×10^−07^	8.03×10^−08^
10	FNDC5	244618	252995	2.53×10^−07^	4.58×10^−09^	3.63×10^−06^	2.13×10^−08^
11	CDH2	325182	1000	9.87×10^−07^	4.86×10^−07^	2.95×10^−08^	1.41×10^−04^
12	OLFM1	52076	10439	3.29×10^−08^	2.29×10^−10^	1.26×10^−03^	1.77×10^−08^
13	RCVRN	383188	5957	1.00×10^−07^	4.69×10^−14^	8.68×10^−08^	4.22×10^−03^
14	PTPN13	866702	5783	2.74×10^−08^	9.53×10^−09^	1.26×10^−06^	2.33×10^−07^
15	HLA-DMA	183337	3108	3.76×10^−04^	1.90×10^−07^	1.15×10^−06^	2.84×10^−04^
16	MYL4	461425	4635	8.30×10^−06^	1.03×10^−06^	2.28×10^−06^	1.25×10^−06^
17	SGCA	796258	6442	1.83×10^−07^	1.02×10^−06^	5.97×10^−07^	2.05×10^−08^
18	GAP43	44563	2596	1.03×10^−04^	1.71×10^−05^	3.22×10^−05^	9.14×10^−05^
19	PSMB8*	624360	5696	2.76×10^−03^	1.72×10^−06^	2.00×10^−05^	1.16×10^−06^
20	PMS2L12*	878652	392713	2.25×10^−03^	4.03×10^−11^	3.34×10^−07^	9.49×10^−04^
21	EHD1*	745019	10938	1.13×10^−05^	1.38×10^−09^	2.90×10^−04^	1.45×10^−03^
22	TNNT2	298062	7139	3.20×10^−05^	1.70×10^−05^	1.97×10^−05^	1.91×10^−05^
23	CBX1*	786084	10951	3.86×10^−08^	1.76×10^−03^	1.86×10^−09^	2.85×10^−03^
24	RBM38	814526	55544	2.84×10^−05^	2.50×10^−07^	1.06×10^−09^	2.41×10^−03^
25	TNNT1	1409509	7138	4.90×10^−05^	4.05×10^−07^	5.91×10^−06^	3.63×10^−06^
26	CRMP1	878280	1400	1.12×10^−04^	2.46×10^−11^	4.80×10^−07^	1.90×10^−03^
27	HLA-DQA1	80109	3117	1.46×10^−03^	1.80×10^−05^	2.67×10^−05^	2.64×10^−04^
28	DPYSL4	395708	10570	7.62×10^−04^	1.63×10^−10^	3.05×10^−06^	5.67×10^−05^
29	PIM2	1469292	11040	2.74×10^−03^	6.31×10^−06^	9.72×10^−06^	1.36×10^−05^
30	CTNNA1	21652	1495	1.09×10^−05^	5.03×10^−11^	1.13×10^−06^	8.77×10^−04^
31	SELENBP1	80338	8991	2.12×10^−07^	2.77×10^−10^	1.46×10^−08^	7.56×10^−04^
32	ELF1	241412	1997	7.03×10^−04^	4.75×10^−05^	5.19×10^−06^	9.91×10^−04^
33	KIF3C	784257	3797	4.80×10^−03^	8.36×10^−11^	1.90×10^−05^	3.69×10^−05^
34	GYG2	43733	8909	9.70×10^−07^	5.56×10^−08^	8.77×10^−06^	1.03×10^−05^
35	LSP1*	143306	4046	1.22×10^−05^	4.34×10^−10^	4.77×10^−04^	6.24×10^−07^
36	MT1L	297392	4500	2.20×10^−03^	2.50×10^−06^	1.84×10^−04^	7.29×10^−04^
37	CHD3*	379708	1107	1.99×10^−07^	4.37×10^−09^	1.09×10^−06^	4.37×10^−04^
38	*EST (CDK6)*	295985	1021	3.08×10^−10^	3.26×10^−03^	2.66×10^−06^	1.63×10^−04^
39	TNFAIP6	357031	7130	7.93×10^−07^	1.92×10^−07^	3.07×10^−06^	1.34×10^−05^
40	WAS*	236282	7454	7.36×10^−04^	7.88×10^−08^	3.27×10^−07^	1.10×10^−03^
41	GAS1	365826	2619	1.72×10^−04^	4.78×10^−11^	4.69×10^−09^	2.04×10^−03^
42	HCLS1	767183	3059	1.42×10^−03^	6.05×10^−06^	3.06×10^−06^	1.66×10^−04^
43	MYO1B	377048	4430	2.41×10^−05^	2.77×10^−15^	5.34×10^−07^	1.71×10^−02^
44	ARPC1B*	626502	10095	1.37×10^−03^	8.58×10^−05^	1.49×10^−04^	1.47×10^−03^
45	HOXB7*	1434905	3217	4.75×10^−07^	4.65×10^−06^	1.80×10^−11^	1.89×10^−02^
46	PRKAR2B	609663	5577	3.77×10^−04^	2.19×10^−05^	4.23×10^−03^	1.16×10^−07^
47	G6PD*	768246	2539	1.67×10^−05^	2.72×10^−03^	7.13×10^−06^	5.18×10^−04^
48	GATA2	135688	2624	3.42×10^−03^	5.37×10^−08^	1.67×10^−05^	1.01×10^−05^
49	CSDA*	810057	8531	3.77×10^−04^	2.10×10^−02^	4.99×10^−10^	7.21×10^−04^
43	MYO1B	308231	4430	3.59×10^−05^	2.84×10^−11^	5.94×10^−06^	1.31×10^−02^
51	EID1*	244637	23741	2.25×10^−03^	4.54×10^−05^	5.89×10^−07^	7.23×10^−04^
52	PSMB10*	68977	5699	1.24×10^−03^	2.80×10^−05^	3.74×10^−04^	1.76×10^−03^
53	FHL3*	796475	2275	2.56×10^−05^	3.26×10^−11^	4.42×10^−07^	5.07×10^−03^
54	ITPR3*	435953	3710	1.31×10^−03^	8.08×10^−05^	1.15×10^−07^	1.22×10^−06^
55	DPYSL2	841620	1808	2.99×10^−05^	4.26×10^−15^	2.83×10^−04^	5.63×10^−07^
56	BIN1	788107	274	2.93×10^−05^	2.53×10^−07^	3.05×10^−03^	1.97×10^−05^
57	PFN2	486110	5217	5.41×10^−03^	1.02×10^−15^	9.16×10^−06^	2.33×10^−02^
58	TLE2	1473131	7089	2.88×10^−07^	5.70×10^−09^	2.24×10^−08^	1.06×10^−03^
59	PGAM2*	283315	5224	3.25×10^−03^	1.21×10^−05^	2.51×10^−02^	5.78×10^−08^
60	ISG20*	740604	3669	9.18×10^−04^	1.04×10^−04^	1.26×10^−04^	7.18×10^−04^
61	RDX*	740554	5962	6.12×10^−07^	4.86×10^−11^	2.57×10^−05^	1.62×10^−03^
62	PPP1R18*	208699	170954	1.94×10^−07^	2.46×10^−06^	3.19×10^−03^	3.19×10^−03^
63	CCND1	841641	595	6.10×10^−04^	1.67×10^−09^	2.10×10^−03^	3.21×10^−06^
64	SMPD1*	729964	6609	3.03×10^−05^	2.00×10^−08^	1.07×10^−04^	2.74×10^−03^
65	MEIS3P1*	450152	4213	3.42×10^−04^	2.82×10^−10^	7.84×10^−06^	3.17×10^−03^
66	MYH10*	823886	4628	2.77×10^−03^	7.30×10^−13^	4.38×10^−06^	1.72×10^−02^
67	IFITM3	809910	10410	1.45×10^−03^	2.50×10^−11^	6.00×10^−09^	5.59×10^−04^
68	ARSB*	502055	411	2.91×10^−04^	9.78×10^−10^	2.01×10^−02^	4.32×10^−03^
69	BCKDHA*	740801	593	1.62×10^−05^	4.15×10^−11^	9.91×10^−06^	1.08×10^−02^
70	NF2*	769716	4771	3.40×10^−04^	3.62×10^−07^	6.50×10^−09^	9.26×10^−08^
71	CLEC3B*	345553	7123	1.24×10^−03^	2.45×10^−04^	1.07×10^−03^	2.73×10^−03^
72	HSPB2	324494	3316	1.77×10^−02^	1.37×10^−09^	2.10×10^−09^	1.74×10^−06^
73	NFKB1*	789357	4790	2.05×10^−05^	4.94×10^−05^	1.92×10^−05^	1.31×10^−02^
74	GNA11*	221826	2767	4.08×10^−07^	1.12×10^−14^	8.04×10^−04^	8.58×10^−03^
75	IGF2	245330	3481	7.93×10^−04^	4.43×10^−04^	8.16×10^−04^	7.51×10^−04^
76	APLP1	289645	333	1.39×10^−03^	1.10×10^−11^	1.18×10^−05^	8.82×10^−08^
77	NFIC*	265874	4782	1.18×10^−04^	3.84×10^−09^	2.50×10^−05^	1.90×10^−02^
78	TEAD4*	346696	7004	1.61×10^−02^	1.37×10^−09^	1.92×10^−05^	9.80×10^−06^
79	HLA-DPB1	840942	3115	3.38×10^−02^	7.46×10^−06^	4.75×10^−05^	8.64×10^−04^
80	HMGA1*	782811	3159	3.37×10^−04^	7.05×10^−05^	5.88×10^−04^	8.40×10^−07^
81	MEST*	898219	4232	1.06×10^−05^	2.25×10^−06^	5.69×10^−05^	1.20×10^−06^
82	PTPN12*	774502	5782	1.32×10^−07^	4.28×10^−07^	2.82×10^−05^	9.16×10^−05^
83	IGLL1*	344134	3543	2.55×10^−03^	7.44×10^−05^	7.19×10^−06^	3.55×10^−02^
84	PTTG1IP*	505491	754	5.37×10^−06^	1.63×10^−12^	4.99×10^−03^	1.83×10^−03^
85	AKAP7*	195751	9465	9.97×10^−04^	1.06×10^−02^	9.20×10^−04^	1.05×10^−03^
86	SERPINH1*	142788	871	1.73×10^−02^	3.87×10^−11^	3.42×10^−04^	1.14×10^−04^
87	SEPT4*	66714	5414	8.07×10^−04^	3.62×10^−07^	2.87×10^−04^	5.90×10^−05^
88	CITED2*	491565	10370	5.18×10^−09^	9.60×10^−10^	8.45×10^−04^	1.00×10^−05^
89	TXNRD1*	789376	7296	3.23×10^−03^	3.25×10^−02^	2.39×10^−06^	6.32×10^−07^
90	*EST (RND3)*	784593	390	1.92×10^−06^	1.30×10^−13^	1.51×10^−04^	7.13×10^−04^
91	TRIP6*	811108	7205	3.74×10^−07^	4.99×10^−10^	3.88×10^−08^	3.27×10^−02^
92	*EST (YAP1)*	308163	10413	3.09×10^−05^	7.70×10^−16^	4.73×10^−09^	8.68×10^−04^
93	TAF15*	1474955	8148	6.55×10^−03^	1.38×10^−05^	2.34×10^−03^	3.45×10^−02^
94	RXRG*	358433	6258	2.31×10^−02^	1.70×10^−08^	5.13×10^−07^	2.87×10^−04^
95	SERPING1	756556	710	2.82×10^−03^	2.01×10^−09^	2.45×10^−05^	7.04×10^−03^
96	MYL1*	628336	4632	1.74×10^−03^	1.72×10^−03^	2.51×10^−02^	1.51×10^−03^

*indicates complement genes. Truncated p-value is the product of p-value of the gene expression value by its rank order in the GANN model and subsequently adjusted using Benjamini and Hochberg false discovery rate.

### Interactome Network Map

The concept of the interactome network map in which the internal organization and functional regulation of cells can be presented using network/graph theory was initially set out by Barabási and Oltvai [8]. In the network map, a single gene is symbolized by a *node,* and the link between genes is known as an *edge,* which can be presented with an arrow to indicate the direction of the link from a *source* node to a *target* node.

Interactome network maps have been used to demonstrate interactions between biological components. These originally utilized off-the-shelf or publicly available modeling tools to analyze associations between biological molecules [9]–[13], but later used customized data mining tools to comprehensively model the interaction between biological molecules. These customized regulatory networks were initially Boolean-based [14], [15], then evolved to Bayesian probability [16]–[18], dynamic ordinary differential equations (ODEs) [19] and in recent years, ANNs [20]–[25]. These and other approaches have been reviewed elsewhere [26]–[30]. An ANN network inference approach was chosen to model the interactions between sarcoma-related microarray genes for the SRBCTs dataset in this study. For reference, we refer to this approach as Artificial Neural Network Inference (ANNI).

### Artificial neural network inference (ANNI)

ANNs have been extensively used for biomarker identification and classification [31]–[37] due to their ability to cope with complexity and nonlinearity within the biology datasets. These features enable ANNs to address a particular question by identifying and modeling patterns in the data [38], [39]. The underlying structure of the multilayer perceptron (MLP) is a weighted, directed graph [24], interconnecting artificial neurons (i.e. nodes) organized in layers with artificial synapses (i.e. links) which carry a value, (i.e. weight) transmitting data (i.e. signals) from one node to the other nodes. All incoming signals from the input layer will be processed based upon a set of defined parameters (i.e. error computation function, acceleration measure, input weights) by the nodes in the intermediate layer (i.e. hidden layer) and an activation function is applied to the resulting sum. This sum is then used to determine the output result (i.e. predicted value) generated by the nodes in the output layer. Due to the connectionist computation in ANNs, the architecture of the ANN can be easily modified to address different questions and able to compose complex hypotheses that can explain a high degree of correlation between features without any prior information from the datasets. Hence, a backpropagation MLP was chosen as ANN to model the gene-gene interaction in this paper.

This study hypothesized that the expression (i.e. up- or down-regulation) of a biomarker can be explained using the remaining biomarkers in the gene pool, if these biomarkers are able to explain one particular categorical outcome (i.e. a disease status). Herein, we explored the influences of all biomarkers among themselves and provide a complete view of all of the possibilities of network interactions for all biomarkers. Therefore, the principle of the algorithm is to show the relationship between genes from the same pool, to shed light on how these molecules interact with each other and to identify new relationships between these molecules by iteratively calculating the influence that multiple variables may have upon a single one. The main advantage of this algorithm lays in its multi-factorial consideration of each input which allows the magnitude of interaction (i.e. inhibitory, stimulatory, bi- or unidirectional) of a given pair of parameters to be determined on the basis of a matrix of full interaction, and by iteratively examining the weights and prediction performance of each single input expression from all the others within the set.

**Figure 1:**
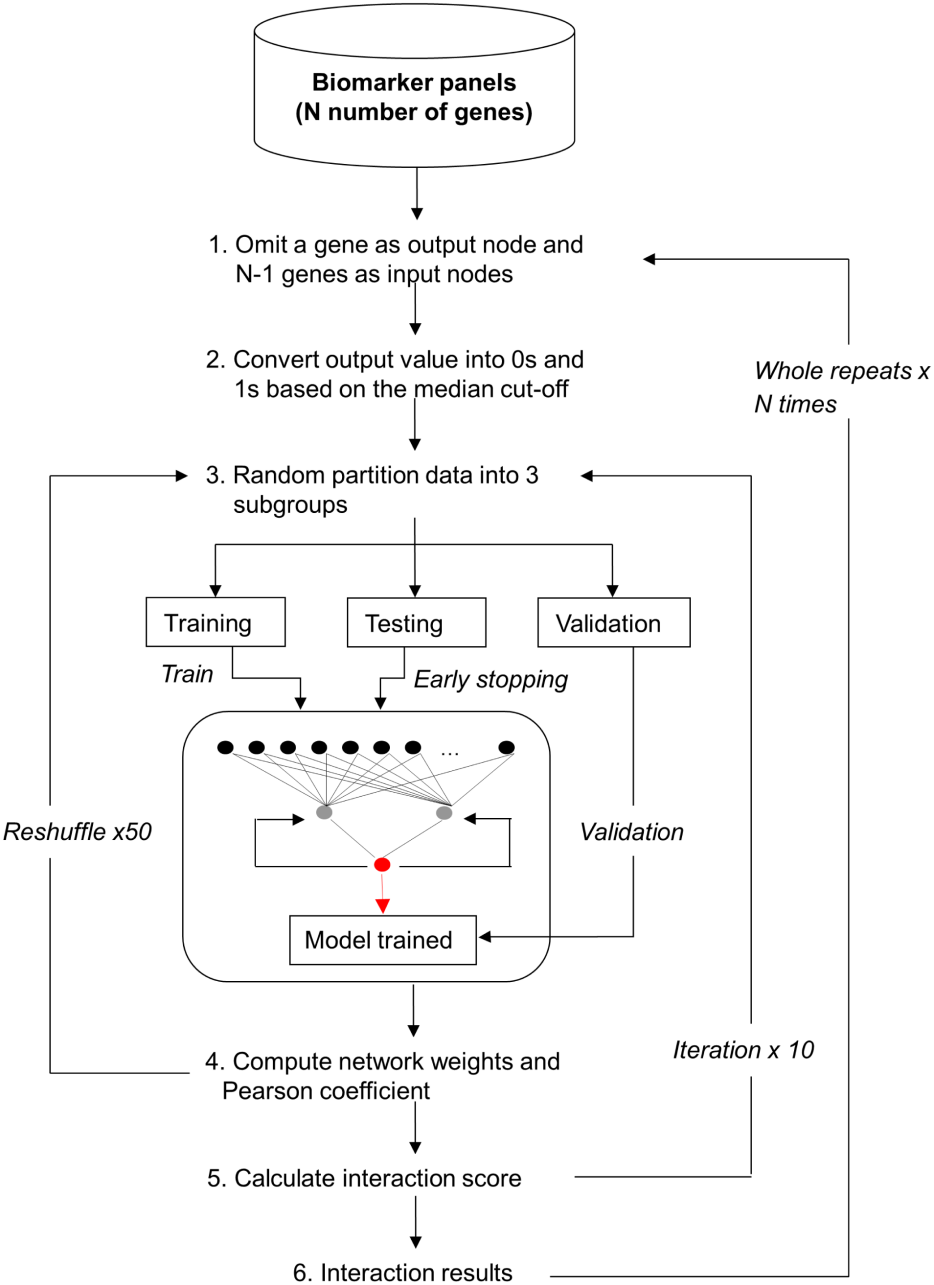
Overview of the interaction algorithm.

**Figure 2:**
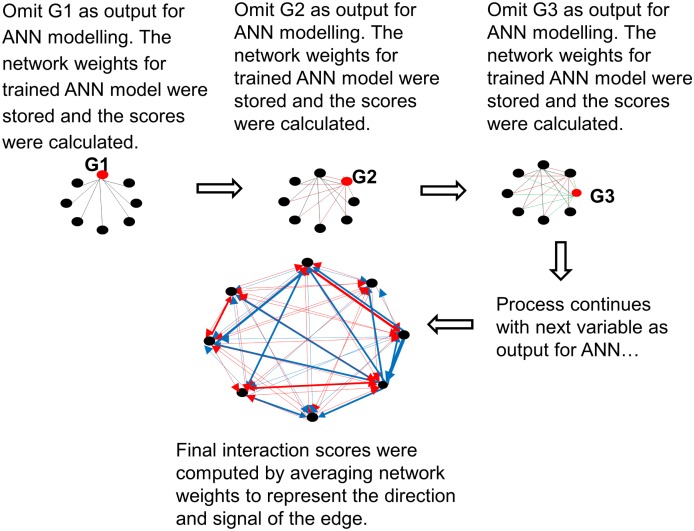
Diagrammatic representation of the interaction algorithm.

Figures 1 and 2 present a schematic and a diagrammatic representation of the interaction algorithm, respectively. A summary of the parameter settings for the algorithm is depicted in Table 2.

**Table 2:** Summary of the interaction algorithm parameters.

Parameter	Setting
Architecture	N-2-1, where N = total number of genes −1
Learning algorithm	Backpropagation
Activation function	Sigmoid
Epochs	300
Threshold of mean squared error (MSE)	0.01
Window of MSE	100
Momentum	0.5
Learning rate	0.1
Pearson *r* cutoff	0.7
Cross-validation	Monte Carlo cross-validation
Random reshuffle	50 times
Ratio (%) for training: testing: validation	60∶20∶20
Number of time the whole process repeatfor single variable	10

#### Multilayer perceptron (MLP)

A 3-layered MLP with backpropagation learning and sigmoid activation function was applied to model gene-gene interactions for sarcoma cancers in SRBCTs data. To prevent any relationships being omitted in the interaction analysis, iterative calculation of the influence that multiple genes may have on a single gene was performed in the following way: The process begins with the first input gene in the gene pool, which was defined as an output node in the ANN and omitted from the pool. The remaining genes were then used to predict the omitted gene, and the weights of the trained ANN model were stored. This process was repeated by omitting the second input gene as an output node in ANN and the remaining genes as the input nodes in ANN and so on. The process was iterated until all the genes in the dataset were used as an output. The average values of these iterations were then computed as the interaction score values.

#### The learning process

Given the initial connection weight 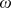 is randomized in-between range −1 to 1, 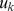 is the sum linear output value from the input signal *x* of node *k* and *b* is the bias value, the input signal *x* and the output signal *y* of node k can be expressed as follows:
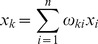
(1)


and
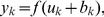
(2)where 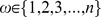 is the connection weights of the input signals 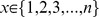 and *f(·)* is the sigmoid activation function which can be defined as:
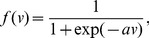
(3)where *v* is the local signal in the node and *a* is the slope parameter of the sigmoid function.

In the iterative learning cycle, the weights are adjusted based on the output error 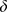 (see Eq. 4), the total sum-of-squares error based on the difference between the network output *y* and the target output *d* of the sample case. In Eq. 4, *N* is the total number of sample patterns and s is the sample pattern.
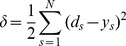
(4)


If both the network output and the target output are identical, no adjustment on the weight in the current learning cycle *T* is required for this sample pattern and a new sample pattern is fed into the network and the learning cycle continues. If the match is not perfect, the adjustment of the weight 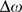 is the proportion of the input signal *x_i_* of node *k*, the learning rate 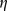 and the size of the error 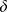:
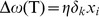
(5)


The new weight for the next learning cycle *T+1* can then be written as:
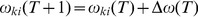
(6)


To reduce the chance of trapping in endless learning cycle, a momentum 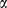 is applied in the learning process and thus, the Eq. 6 can be rewritten as:
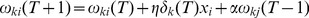
(7)


#### Interaction

To define an interaction map for the genes, the weights of the trained ANN models were used to illustrate and score the interaction between genes, such as the intensity of the relationship between a source gene and a target gene, and the nature of the relationship either stimulatory or inhibitory (i.e. positive or negative sign in correlation result, respectively). The Pearson correlation coefficient *r* with a cutoff value of 0.7 was implemented in the algorithm to remove the least significant interaction scores.

#### Monte-Carlo cross-validation (MCCV)

To prevent the ANN model from being over-trained, the MCCV strategy was applied as follows. The sample set was randomly partitioned (without replacement in the sample set) into 3 smaller groups: training, test and validation, with the ratio of 0.6∶0.2∶0.2, respectively. The training and test sets were used to train the ANN model and the validation set was used to examine the trained model. After the model was trained, all samples were re-shuffled and re-partitioned into training, test, and validation sets. This re-shuffle and re-partition process was repeated 50 times, and each time, new different sets of training, test and validation samples were randomly generated.

The entire process cycle was repeated 10 times for single gene. The algorithm was coded in C and empirical work on simulated dataset was presented in the subsequent section.

### Visualization of interactome network maps

The Cytoscape software platform (version 2.8) for molecular interaction display was used in this study. Cytoscape [40], [41] is an open-source software for analyzing complex biological networks by visually interrogating the relationship of their components using a variety of plug-ins.

### Assessment of ANNI using a simulated dataset

A simulated dataset has been generated to assess the prediction ability and the robustness of the optimum ANN parameters (see Table 2) for correctly identify correlated features and to compute their correlation values. This dataset was created using R and contains 100 samples with 25,000 features in which 32 were highly correlated, distributed into 2 major classes. Amongst these 25,000 features, 100 best predictive including 29 pre-defined correlating features were pre-selected using a data mining algorithm.

Despite the obvious advantages of using well-characterised simulated datasets for the testing of new analysis tools, it is important to note that human biological data are complex and the lack in the knowledge of actual biological correlation between sample replicates, molecular relationship between a biological state of a cell and transcript expression, biochemical reaction mechanisms underlying regulatory interactions between features and activity changes from one state to another. This makes artificial data valuable for algorithm development, but is not of value for comparing different methods.

To assess the predictive ability of the algorithm, criteria such as number of hidden nodes used in the network, correlation analysis comparing the predicted correlation scores for each pair of the features with their actual correlation values, interaction signs analysis comparing between the sign of the actual correlation value and the sign of the predicted interaction score and true positive rate (TPR) have been considered. Table 3 shows a summary of the results.

**Table 3:** Summary of the assessment results.

No. of hidden nodes	No. of features	Person’s coefficient	% of correctly assigned signs	Ave. true positive rate (%)
2	32	0.805	89.16	93.16
	100	0.865	91.26	70.33
5	32	0.653	80.31	89.00
	100	0.871	90.89	68.50
10	32	0.607	79.32	92.66
	100	0.866	88.55	81.16

High accuracy on the TPR, correlation result and predicted interaction sign confirm the feasibility of this approach to accurately identify the simulated features having strong correlations. In terms of network architecture, there is no significant improvement on TPR when the number of hidden nodes increases, thereby suggesting that the number of hidden nodes does not affect the predictive ability of the algorithm. A model with 2 hidden nodes performs equally good, or better than those equipped with higher number of hidden nodes and lesser computational time is needed to process the query. Thus, 2 hidden nodes were implemented in the algorithm.

A full, comprehensive empirical validation on the algorithm can be found in Lemetre’s PhD Thesis [42].

## Results and Discussion

**Figure 3:**
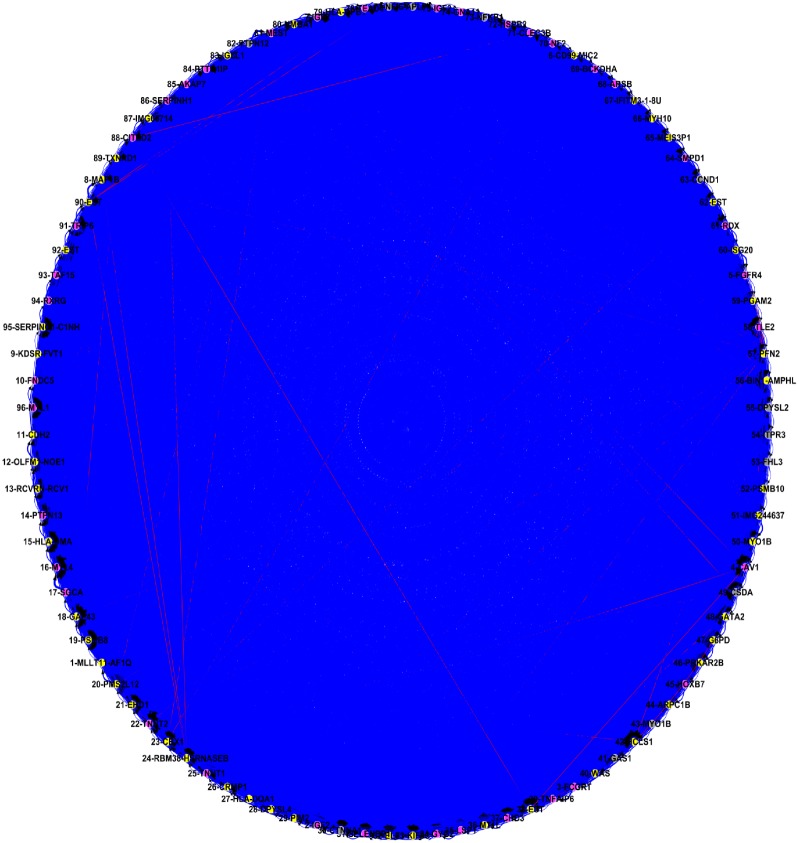
A complete interactome network map.

**Figure 4:**
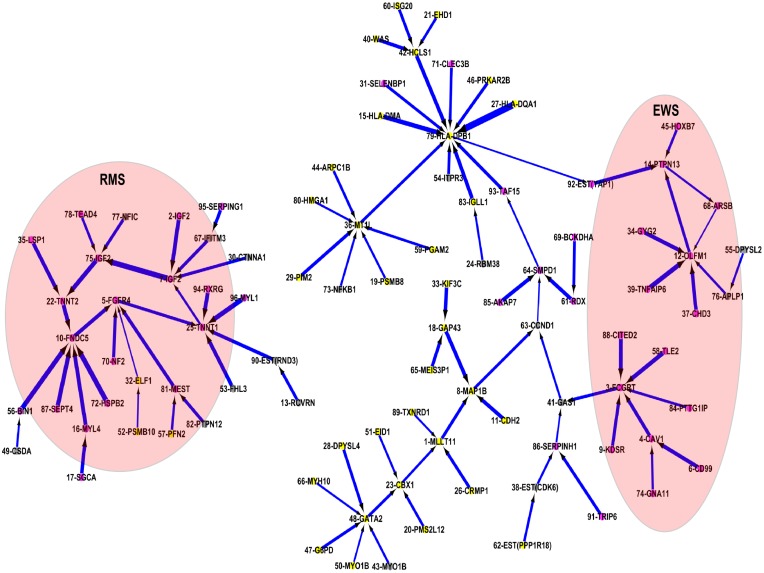
A simplified interactome network map for the 96 selected genes by ANN network inference algorithm. The red node is the genes with high expression values in either of the sarcoma cancers. The gray node is the gene with high expression values in more than one cancer groups in which one of these groups is sarcoma cancer. Yellow node is the genes with low expression values in both sarcoma cancers.

Analysis of all 96 genes from the interaction analysis produced a matrix of (96×(96–1)) 9,120 potential interactions (Figure 3). Due to the high dimensionality and complexity of the interactions between all genes, it clearly appeared that no relevant information could be elucidated in the map. Thus, only the 96 strongest associations (based upon the averaged values of the scores leading from a given input to the output), each associated to one of the 96 genes, were imported in Cytoscape and are presented in Figure 4. The consequence of this is that for each of these 96 genes, only the strongest interaction among all interactions with the other 95 genes was modeled. This greatly simplified the map, and facilitated the interpretation and understanding of the key features within the map. Table 1 presents the list of 96 genes with its truncated p-values based upon the product of the p-values and its rank order in the GANN model. These truncated p-values are adjusted using Benjamini and Hochberg false discovery rate procedure [43].

Genes (i.e. target genes) with most associations include MT1L, HLA-DPB1, GATA2, OLFM1, FCGRT, TNNT1, and FNDC5. The repetitive genes in the map were IGF2 in ranks 2, 7, and 75; and MYO1B in ranks 43 and 50. These repetitive genes show consistent expression pattern in which all 3 selected IGF2 genes show high expression values and strong negative interactions in RMS, and the 2 selected MYO1B genes are in non-sarcoma group. Low truncated p-value (stringent p-value<0.005) for RMS- and EWS-regulated genes are observed in Table 1.

RMS and EWS are soft tissue sarcomas that can be found virtually anywhere in the body and share common clinical characteristics, more frequently occurring in males than females and normally found in children. Early-stage RMS patients are often confused with EWS, consequently inducing lymphatic-related cancer when the RMS tumor migrates to lymph node. Although these tumors have similar clinical conditions, EWS is commonly developed in bones, whereas RMS is more frequently found in skeletal muscle. In the map, genes which have exhibited an up-regulation in both RMS and EWS groups include CTNNA1, GAS1, IFITM3, YAP1, SERPING1, CSDA, FHL3, NFIC, TRIP6, TAF15, and RXRG.

CSDA is a repressor gene involved in various biological processes including skeletal muscle tissue development and organ growth. FHL3 is only expressed in skeletal muscle and could be involved in tumor suppression and repression of MyoD expression. IFITM3 is IFN-induced antiviral protein that plays a role in innate immune response to virus infections. NFIC is a cellular transcription factor involved in DNA binding transcription factor activity. Although the function of TRIP6 is not fully understood, it has been associated with ligand binding of the thyroid receptor in the presence of thyroid hormone. TAF15 plays specific roles during transcription initiation in RNA binding and it may be involved in protein-protein-interaction. RXRG is a retinoic acid (RA) receptor that regulates gene expression in various biological processes, including skeletal muscle tissue development, heart development, and response to hormone stimulus.

### Rhabdomyosarcoma (RMS)

RMS is a connective tissue related cancer. The cause of this sarcoma is unknown, but its development has been associated with desmin, MyoD1 and myogenin (MYOG) [44]. MyoD1 is a regulator involved in muscle regeneration and muscle cell differentiation and MYOG is the muscle-specific transcription factor that plays a role in the development of functional skeletal muscle.

TNNT1 (mainly expressed in heart muscle) and FNDC5 (normally induced by the expression of PGC-1 alpha in muscle) are two highly associated genes in the RMS cancer, which act as target genes (i.e. interaction hubs) that interconnect genes which also show high expression values in the RMS cancer. These 2 genes are bridged by FGFR4, which plays a role in the regulation of cell proliferation, differentiation and migration.

#### TNNT1 interaction cluster

TNNT1 protein is inhibited by genes RXRG, MYL1, RND3, FHL3 and FGFR4. Amongst these genes, RXRG, MYL1 and FHL3 were complementary genes to the genes reported by Khan *et al*.

RXRG is a tumor suppressor gene that mediates the anti-proliferative effect of retinoic acid (RA), an essential metabolite of vitamin A for the growth, development and cell differentiation of vertebrate species. This protein suppresses tumor growth by increasing the anti-proliferative effects of RA in the tumor cells. MYL1 is the motor protein known for the role in muscle cell activities including vesicle transportation inside the muscle cell. A negative interaction on this protein indicates that tumor cells are constrained in a particular location rather than move randomly. FHL3 expressed only in skeletal muscle has been known by its role in skeletal myogenesis [45]–[47], although its actual function is unknown. This gene has been related to cell spreading and actin stress fiber disassembly [46] and is involved in tumor suppression/repression of MyoD expression. RND3 is a member of the Rho family GTPase protein superfamily that acts as a negative regulator in cytoskeletal organization. It is known to have a role in myoblast fusion [48] and to be responsible for down-regulation in focal adhesions and stress fibers. FGFR4 is a tyrosine kinase receptor responsible for signal transduction activities in the cell. Although the activity of this protein is undetectable in normal tissues, it becomes active when a tumor is formed. High expression of FGFR4 has been associated with advanced-stage in RMS cancer and a poor survival rate [49].

Although over-expression of FGFR4 in the map (see Figure 4), is inhibited by other low/moderately expressed genes, it may suggest the formation of tumor cell in skeletal muscle. Over-expression of FGFR4 may stimulate the expression of TNNT1; the regulator for striated muscle contraction, despite the fact that a negative interaction between these 2 genes was detected. Low expression of FHL3, RXRG, MYL1 and RND3 may also promote the mutation of TNNT1 genes due to a suppression of the anti-proliferative effects of RA in the RMS cells. The low activity of these genes might influence normal cell spreading, actin stress fiber disassembly and, consequently, tumor cells migration. TNNT1 is the diagnostic marker for nemaline myopathy [50], [51]. The high expression value in mutated TNNT1 genes suggests that RMS tumors may be congenital.

#### FNDC5 interaction cluster

FNDC5 protein expressed in muscle is inhibited by RMS-expressed genes including TNNT2, BIN1, SEPT4, MYL4 and HSPB2. Up-regulation of this gene suggests that the increased level of irisin hormone [52], [53] promotes the beneficial effects of exercise on metabolic pathways. TNNT2 and BIN1 are proteins that play important roles in cardiac muscle development. Over-expression of TNNT2 has been correlated to myocardial stunning in hemodialysis patients [54], [55], and the disruption of this gene could lead to impaired cardiac development in the embryo and infant [56]–[58]. Deficiency of the BIN1 gene has been correlated with cardiomyopathy. SEPT4 is the nucleotide binding proteins highly expressed in heart and brain. It regulates cytoskeletal organization during embryonic and adult life [59]. MYL4 is the hexameric ATPase cellular motor proteins that commonly found in embryonic muscle and adult atria. Over-expression of this gene was normally found in patients with hypertrophic cardiomyopathy and congenital heart diseases [60]. HSPB2 is another protein that expressed in the heart and skeletal muscles. Over-expression of this gene indicates the efficient recovery of motor neurons following nerve injury [61].

In the map (Figure 4), observation on the over-expression pattern on TNNT2, BIN1 and MYL4, moderately expression on HSPB2 and SEPT4 is lowly expressed. This suggests that RMS patients have low chance to develop cardiomyopathy due to high expressions of TNNT2, BIN1, SEPT4 and HSPB2 in RMS pathway could potentially suppress expression level of FNDC5.

### Ewing’s sarcoma (EWS)

EWS is a bone malignancy which commonly affects areas including the pelvis, femur, humerus and the ribs [44]. The cell origin of this tumor is uncertain. However, this cancer has a shared cytogenetic abnormality with the primitive neuroectodermal tumor (PNET) which arises from the soft tissue or bone. The shared cytogenetic abnormality involves the EWS/FLI fusion in t(11;22)(q24;q12), a signature marker for EWS/PNET from other small round tumors [62].

Two of the highest associated genes observed were FCGRT and OLFM1 (Figure 4). FCGRT and OLFM1 acted as interaction hubs, in which FCGRT plays a role in interconnecting genes associated to the EWS/FLI fusion and OLFM1 integrates variety biological process performed by other genes.

#### FCGRT interaction cluster

FCGRT protein is known as the promoter marker to EWS-FLI fusion, one of the common cytogenetic abnormalities on the t(11;22) translocation for Ewing tumors. In the map, highly expressed FCGRT was suppressed by TLE2, CITED2, CAV1, PTTG1IP and KDSR. Amongst these associated genes, CITED2 and PTTG1IP were in addition to those genes reported by Khan *et al*.

CITED2 is a cardiac transcription factor responsible for inhibiting transactivation activity of hypoxia-induced genes. Mutation of this gene decreases its ability to mediate the expressions of VEGF and PITX2C genes, suggesting that it may play a role in the development of congenital heart disease [63]. Over-expressed CITED2 induces resistance to cisplatin [64], a commonly used chemotherapeutic drug for sarcomas and other solid malignancies. CAV1 is another promoter marker associates to EWS-FLI fusion. Over-expression of this gene promotes metastasis on Ewing tumor [65], [66]. PTTG1IP is the promoter gene that has been correlated with the Runx2 gene expression in osteoblastic differentiation and skeletal morphogenesis [67]. Runx2 is the master regulator of osteoblast differentiation and study reviewed that blockade of this gene by EWS-FLI induce osteoblast specification of a mesenchymal progenitor cell and thus, disrupting interactions between Runx2 and EWS-FLI may promote differentiation of the tumor cell [68]. TLE2 proteinTLE2 is the member of TLE family and its actual function is not well known. It is believed that the expression of TLE family members is required for EWS oncogenic transformation [62] and acts as repressor protein in histone modification when recruited by NKX2–2 gene [62], [69]. NKX2–2 is the known immunohistochemical marker for Ewing sarcoma [70]. KDSR is the murine gene and has been known by its role in the development of spinal muscular atrophy (SMA) disease in cattle [71]. Although its actual function in human SMA is unknown, a study has claimed that the mutation of this gene does not contribute significantly to the cause of human SMA [72], rather that it associated with chromosomal rearrangement in chronic lymphatic leukemia [73], [74].

In Figure 4, over-expression of CAV1, KDSR and CITED2 inhibited up-regulated FCGRT suggests these EWS patients are EWS-FLI affected sarcoma and have deficiency of cisplatin controlled repression of EWS-FLI fusion. Low expression values of TLE2 and PTTC1IP further indicate the promotion of tumor cell differentiation and histone modification due to inhibition on Runx2 and NKX2–2 genes.

#### OLFM1 interaction cluster

OLFM1 protein which has abundant expression in brain has been associated to the biological process in nerve tissue, is inhibited by GYG2, APLP1, CHD3, TNFAIP6, and ARSB genes. This cluster shows the interaction between wide ranges of glycoproteins, histone deacetylase and Wnt signaling pathway. GYG2 and APLP1 are glycoproteins expressed in liver and membrane, respectively. GYG2 is a self-glucosylating protein found in chromosome X and involved in the initiation reactions of glycogen biosynthesis and blood glucose homeostasis. Over-expression of this gene resulted in excess glycogen storage levels in liver and may lead to glycogenosis due to the deficiency of liver phosphorylase. APLP1 is a membrane-associated glycoprotein that is cleaved by secretases and up-regulation of this protein reduced endocytosis of amyloid precursor protein (APP), and makes more APP available for alpha-secretase cleavage [75].

ARSB is a sulfatase protein responsible for protein fragmentation and mediating intracellular oxygen signaling. Deficiency of this gene due to hypoxia leads to an accumulation of GAGs protein in lysosomes, resulting to mucopolysaccharidosis [76]. CHD3 is part of a histone deacetylase complex participating in chromatin remodeling. Histone deacetylase removes acetyl group on a histone so that the DNA can be tightly wrapped by histones. TNFAIP6 is a multifunctional protein that exhibited in many pathological and physiological contexts. It plays important roles in inflammation and tissue/bone remodeling. It acts as a protector by suppressing tumor necrosis factor (TNFSF11)-induced bone erosion caused by inflammatory processes in arthritis diseases, and exhibits a homeostatic function by interacting with bone morphogenetic protein 2 (BMP2) and TNFSF11 to balance mineralization by osteoblasts and bone resorption by osteoclasts [77]. The binding of TNFSF11 with GAGs and other proteins ligands enhances its activity in various biological processes, including leucocyte adhesion and anti-plasmin activity [78].

OLFM1 protein has been associated with Wnt signaling pathway in which the activation of Wnt signaling pathway in receptive endometrium suppresses the OLFM1 gene and leads to ectopic pregnancy in humans [79]. Wnt signaling pathway plays a critical role in the normal development of multiple neuroectodermal tissues, cell degradation and cell death. This pathway has been associated with neuroectodermal Ewing sarcoma [80], osteosarcoma [81] and possibly embryogenesis [82].

In the map (Figure 4), up-regulation of the OLFM1 gene suppresses the activities of extracellular inhibitors in Wnt signaling pathway. This may promote tumor cell proliferation, learning impairment, suffocation of normal cells (i.e. hypoxia) in ARSB and a deficiency of APP for alpha secretase cleavage in EWS patients. Alpha secretases have been implicated in the regulation of learning and memory formation. Furthermore, over-expression of CHD3 in tumor cells accelerates the interactions between DNA and nuclear proteins, and contributes to tumor cell differentiation, proliferation and migration. Although the moderately expressed genes GYG2 and TNFAIP6 suppress the activity of tumor proliferation, intervention from over-expressed CHD3 and down-regulated APLP1 and ARSB promote diversification of Ewing tumors.

A triangular affair between Wnt signaling pathways, Fas/Rho signaling pathways and intracellular oxygen signaling is observed in the map. Down-regulated ARSB (mediates oxygen in intracellular process) due to intervention of moderately expressed Fas receptor PTPN13 (a large intracellular protein mediates programmed cell death and regulates Rho signaling pathway) fails to activate inhibitors in Wnt signaling pathways (over-expression of OLFM1). Both the Fas receptor and Wnt signaling pathways mediate programmed cell death and the Rho signaling pathway responsible for cell proliferation, apoptosis and gene expression. The over-expression of PTPN13 was suppressed by OLFM1.

## Conclusions

We have previously shown that assessing the statistical significance of genes, based on classification accuracy alone, may not be useful for cancer diagnosis, as classification results are subject to the proposed computational methods and a lack of biological association to those genes to provide a global view on the development of the tumor of interest. Thus, this paper discussed the feasibility of using ANNI to model the potential interactions between the correlated genes in childhood sarcoma cancers. This interaction information could be useful for other researchers who are assessing the practicality of using these genes as new target markers for sarcoma cancers in the clinical setting.

Due to the high dimensional and complexity of the interactions between genes, this paper showed only the strongest association for each of the genes, rather than the complete potential 9,120 interactions existing among them. Even so, this approach highlighted that certain genes were highly influential on sarcoma cancers.

Based on the interaction map, several observations on sarcoma cancers in SRBCTs dataset were noted. The RMS patients in this dataset are likely to be congenital (due to up-regulation of mutated TNNT1 stimulated by FGFR4 and suppression of anti-proliferative effects of RA in FHL3, RXRG, MYL1 and RND3) and these patients have a lower risk of developing cardiomyopathy (due to over-expression in TNNT2, BIN1, SEPT4 and HSPB2 potentially suppress the expression level of FNDC5). The EWS patients are likely to be affected by EWS-FLI fusion (due to deficiency of cisplatin controlled repression, up-regulation of CAV1, KDSR and CITED2, and suppression of TLE2 and PTTC1IP) and involves various signaling pathway complications including Wnt, Fas/Rho and intracellular oxygen pathways (due to over-expression in OLFM1 and CHD3, and low expression in APLP1 and ARSB).

Additional information: The proposed algorithm will be available by contacting the corresponding author. Individuals from all disciplines are invited to access the software on a collaborative basis. Information on the development and background to the algorithm are publicly available in Lemetre’s PhD thesis, which is referenced in the manuscript.
